# The C-Terminal TDP-43 Fragments Have a High Aggregation Propensity and Harm Neurons by a Dominant-Negative Mechanism

**DOI:** 10.1371/journal.pone.0015878

**Published:** 2010-12-31

**Authors:** Chunxing Yang, Weijia Tan, Catheryne Whittle, Linghua Qiu, Lucheng Cao, Schahram Akbarian, Zuoshang Xu

**Affiliations:** 1 Department of Biochemistry and Molecular Pharmacology, University of Massachusetts Medical School, Worcester, Massachusetts, United States of America; 2 Brudnick Neuropsychiatric Research Institute, Department of Psychiatry, University of Massachusetts Medical School, Worcester, Massachusetts, United States of America; 3 Department of Cell Biology, University of Massachusetts Medical School, Worcester, Massachusetts, United States of America; 4 Neuroscience Program, University of Massachusetts Medical School, Worcester, Massachusetts, United States of America; Universidade Federal do Rio de Janeiro, Brazil

## Abstract

TAR DNA binding protein 43 KD (TDP-43) is an essential gene that regulates gene transcription, mRNA splicing and stability. In amyotrophic lateral sclerosis (ALS) and frontotemporal dementia (FTD), two fatal neurodegenerative diseases, TDP-43 is fragmented, generating multiple fragments that include the C-terminal fragment of ∼25 KD. The role of these fragments in the pathogenesis of ALS and FTD is not clear. Here we investigated the aggregation propensity in various polypeptide regions of TDP-43 in mammalian cells and the effect of these fragments on cultured neurons. By expressing the full length and various TDP-43 fragments in motor neuron-derived NSC-34 cells and primary neurons, we found that both N- and C-terminal fragments of TDP-43 are prone to aggregate and the C-terminal end of RRM2 region is required, though not sufficient, for aggregation. The aggregation of the TDP-43 fragments can drive co-aggregation with the full-length TDP-43, consequently reducing the nuclear TDP-43. In addition, the TDP-43 fragments can impair neurite growth during neuronal differentiation. Importantly, overexpression of the full-length TDP-43 rescues the neurite growth phenotype whereas knockdown of the endogenous TDP-43 reproduces this phenotype. These results suggest that TDP-43 fragments, particularly the pathologically relevant C-terminal fragments, can impair neuronal differentiation by dominant-negatively interfering with the function of the full length TDP-43, thus playing a role in pathogenesis in ALS and FTD.

## Introduction

TAR DNA binding protein-43 (TDP-43) has been identified as a major component of the ubiquitin-positive protein inclusions in neurons and glia in ALS, FTD as well as several other neurodegenerative disorders [1]. TDP-43 is a highly conserved, non-redundant gene in metazoan species [2] and is an essential gene because its deletion leads to embryonic lethality [3], [4], [5]. TDP-43 has two RNA binding motifs, RRM1 and RRM2, and binds RNA and numerous other proteins to form one or multiple high molecular weight protein RNA complexes [6], [7], [8]. As an abundant nuclear protein [9], TDP-43 regulates gene expression by modulating transcription, splicing and stability of mRNA [10], [11], [12], [13], [14], [15]. TDP-43 is also present in granular structures in the cytoplasm and may be involved in RNA transport and stress response [6], [16], [17].

In both ALS and FTD, TDP-43 is fragmented, resulting in lower molecular weight C-terminal fragments of ∼35 to ∼25 KD [18]. The C-terminal fragments are prominent in the inclusions in FTD brains and in the insoluble protein fractions from the affected CNS areas of ALS and FTD [18], [19]. These fragments are phosphorylated at serine 409 and 410 and antibodies against this phosphorylation epitope is used as an excellent marker for visualizing TDP-43 inclusions [20]. Recently, these features are reproduced in several in vitro experiments [21], [22], [23] and in transgenic mice that overexpress TDP-43 and display neurodegenerative phenotypes [24], [25], [26], [27]. Despite its potential role in the pathogenesis of ALS and FTD, neither the mechanism whereby the C-terminal fragment is produced nor its effect on cells is clear. The C-terminal fragments may be produced from proteolytic cleavage by caspases [28], although some cleaved fragments from patients do not match the caspase consensus cleavage sites [29], [30]. Progranulin deficiency may stimulate the production of the C-terminal fragments [28] but this remains to be replicated [31]. Expression of the C-terminal fragments in cultured cells generally reproduces the aggregation and phosphorylation [22], [23], [31]. However, the pathological role of the C-terminal fragments remains unclear.

To further understand the aggregation propensity of TDP-43 and the role of TDP-43 fragments in pathogenesis, we engineered a series of TDP-43 truncation fragments and expressed these fragments in cultured neurons. We found that both the C-terminal and the N-terminal fragments of TDP-43 can form aggregates as long as they contain the C-terminal end of the RRM2, which is necessary but by itself is insufficient for aggregation. The aggregation of the TDP-43 fragments can drive co-aggregate with the full-length TDP-43, thereby reducing nuclear TDP-43. Furthermore, the aggregation-prone fragments can impair neurite growth during neuronal differentiation. Importantly, this impairment can be rescued by an overexpression of full-length TDP-43 and can be reproduced by a knockdown of the endogenous TDP-43 expression. These results suggest a model where the C-terminal fragments of TDP-43, found in ALS and FTD patients, harm neurons by a dominant-negative mechanism.

## Results

### Many TDP-43 fragments are prone to aggregate when expressed in mammalian cells

**Figure 1:**
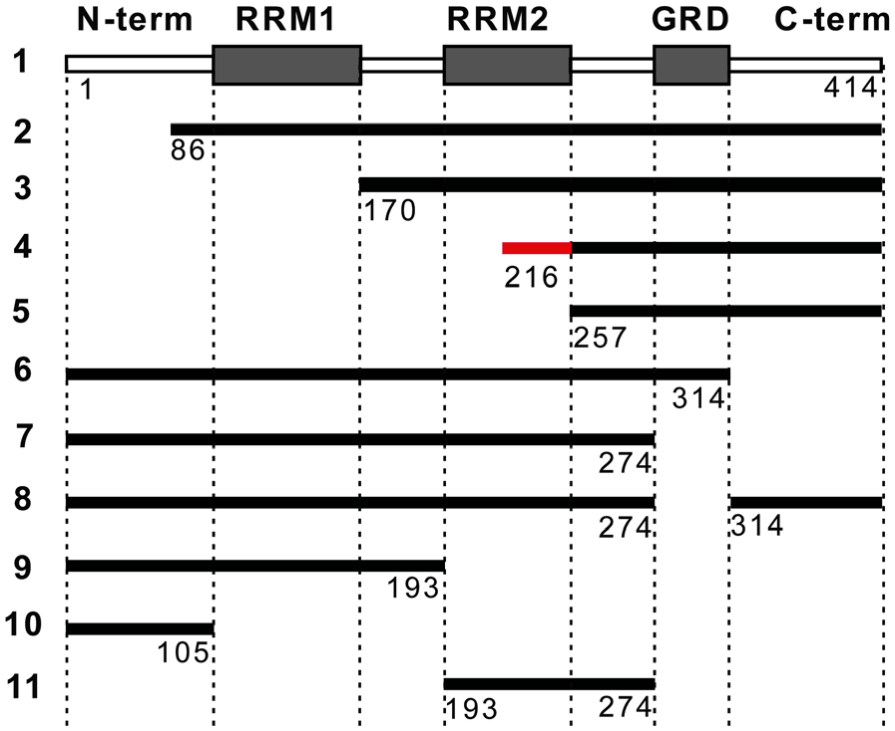
Constructs of TDP-43 fragments. A schematic illustration of various constructs of human TDP-43 fragments. The protein domains are marked on the top. RRM means RNA recognition motif. GRD means glycine-rich domain. The structure is not drawn in proportion. The amino acid numbers are marked for each mutant. The red segment in construct 4 marks the C-terminal half of the RRM2 that is necessary for aggregation (see text). For construct 8, the GRD was deleted and then the two fragments were joined together. EGFP was fused to the C-terminus of all the TDP-43 constructs.

**Figure 2:**
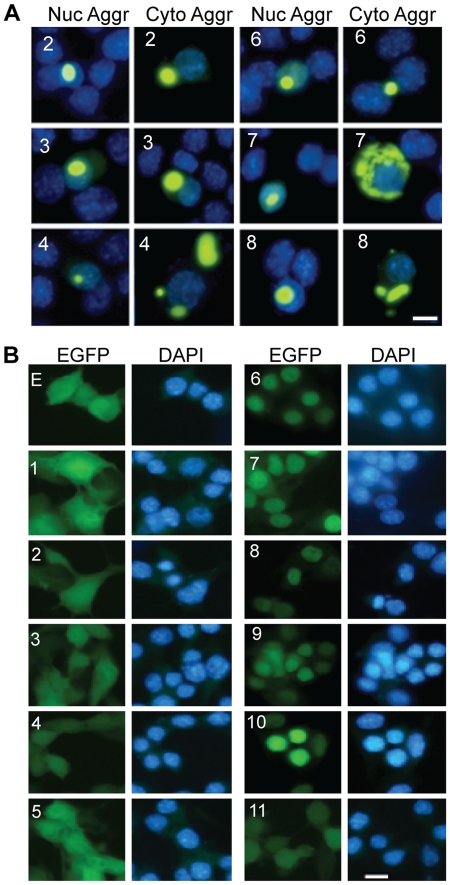
TDP-43 aggregation and distribution of non-aggregated TDP-43-EGFP fragments in NSC-34 cells. (**A**) Examples of cells with TDP-43 aggregates. The numbers in each row indicate the constructs. Both nuclear (Nuc Aggr) and cytoplasmic aggregates (Cyto Aggr) are shown. The nuclei are visualized with DAPI. The scale bar marks 10 µm. (**B**) TDP-43-EGFP distribution in cells without aggregates. The cells were transfected with the constructs: EGFP (E) and various TDP-43-EGFP as marked on each row of panels. The cells were fixed 48 hrs after the transfection and then photographed.

To understand the properties of fragmented TDP-43, we made a series of constructs that synthesize the N-terminal and C-terminal truncated TDP-43 fragments (Fig. 1). We tagged the constructs with EGFP at their carboxyl termini so that we can easily follow the behavior of the proteins synthesized from these constructs. After transfection into motor neuron-like NSC-34 cells [32], we observed intracellular aggregates from several TDP-43 fragments (Fig. 2A). We quantified the percentage of aggregate-containing cells among the GFP-fluorescent cells and also the percentage of cells with aggregates in the nucleus or the cytoplasm (Table 1). We did not observe aggregates from the full-length TDP-43-EGFP fusion protein (Fig. 2B). However, removal of the N-terminal end of TDP-43 up to the middle of the RRM2 (constructs 2, 3 and 4) resulted in aggregates (Fig. 2A; Table 1). Further removal of the C-terminal half of the RRM2 (construct 5) diminished aggregation (Fig. 2B, Table 1). Similarly, removal of the C-terminal end of TDP-43 up to the entire glycine-rich domain (constructs 6, 7 and 8) led to aggregation (Fig. 2A; Table 1). Further removal of RRM2 (constructs 9 and 10) abolished aggregate formation, so did removal of both N- and C-termini of TDP-43 (construct 11; Table 1). These observations were fully replicated in HeLa cells (data not shown) and suggest that C-terminal half of the RRM2 domain is necessary but not sufficient for TDP-43 aggregation.

**Table 1:** TDP-43 aggregation in NSC34 cells.

		Aggregates (%±SE)			
Construct	Soluble protein	%Cells	%Nuc	%Cyto	Total cells counted
1	N	0			
2	N/C	26.3±8.1	10.8±5.1	89.2±5.1	286
3	N/C	34.4±9	10.7±3.8	89.3±3.8	341
4	N/C	44.6±8.9	4.9±3.7	95.1±3.7	347
5	N/C	<1		100	ND
6	N	9.4±0.8	20±7.1	80±7.1	1174
7	N	9.4±2.4	9.9±0.2	90.1±0.2	1101
8	N	17.9±4.5	14.4±4.8	85.6±4.8	620
9	N	0			
10	N	0			
11	N/C	0			

**Figure 3:**
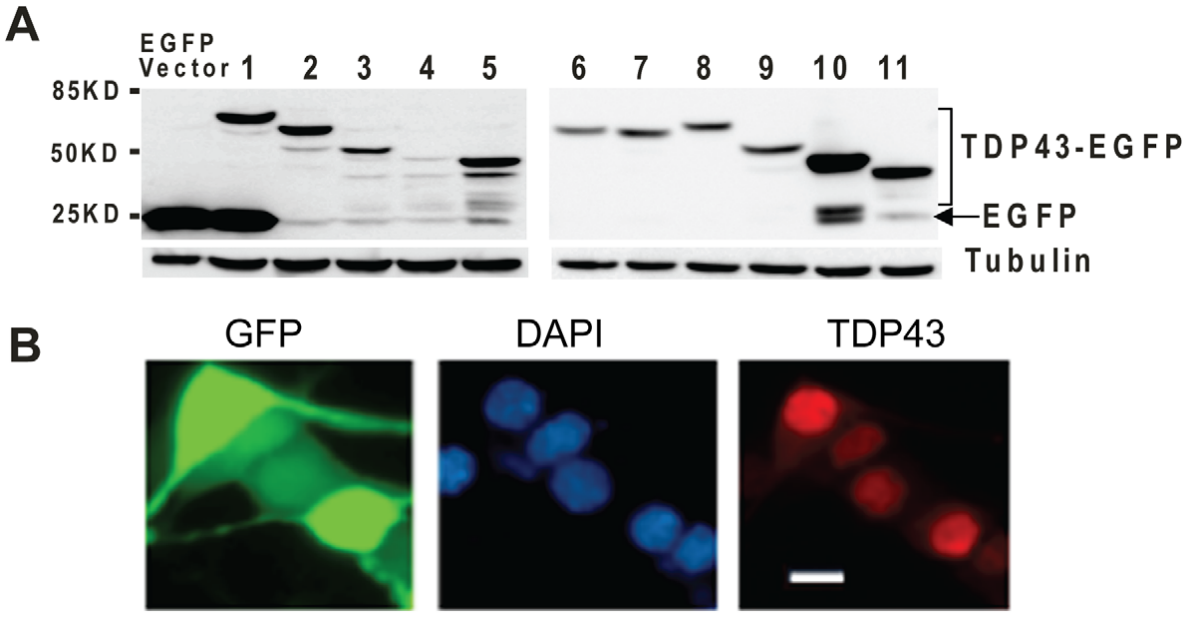
Expression of the various TDP-43-EGFP constructs in NSC-34 cells. (**A**) Forty eight hours after transfection, proteins were extracted from NSC-34 cells and the fusion proteins were detected by Western blot using an EGFP antibody. Fifteen µg of protein was loaded in each lane. α-Tubulin was detected as loading control. Left and right panels are two independent gels. (**B**) Cells was transfected with construct 1 alone and then stained with antiTDP-43 primary and Alexa Fluor®568-conjugated secondary antibodies. The nuclei are stained with DAPI. The scale bar marks 10 µm.

Values were averages of 4 or more experiments. Distribution of soluble TDP-43 fusion proteins was determined in cells that did not contain aggregates. All distribution was determined based on GFP signal except construct 1, which was determined by staining for TDP-43 (see Fig. 3 and the text). Nuc means nucleus; Cyto means cytoplasm; % transfected means the percentage of the transfected cells detected by visualizing GFP; % nucleus means percentage of aggregate-positive cells where the aggregates were located in the nucleus; % cytoplasm means percent of aggregate-positive cells where the aggregates were located in the cytoplasm; SE, standard error.

In general, cells transfected with the C-terminal fragments 2, 3 and 4 had a high percentage of aggregate-containing cells than the cells transfected with the N-terminal fragments 6, 7 and 8 (Table 1). In cells where there were no aggregates, all the C-terminal fragments were predominantly cytoplasmic while all the N-terminal fragments were predominantly nuclear (Fig. 2B, Table 1). This is expected based on the literature that the nuclear localization signal is located in the N-terminal region [33]. Surprisingly, we notice that the full length construct 1 did not show a pattern of nuclear enrichment by visualizing EGFP fluorescence (Fig. 2B). To determine whether the EGFP signal truly reflects the distribution of TDP-43, we examined the protein integrity in the cell extracts using Western blot. When probed by an anti-GFP antibody, all constructs showed a fusion protein band (Fig. 3A). However, construct 1 also showed a strong EGFP band at ∼25 KD, indicating the EGFP signal in the cells transfected with construct 1 largely reflects the distribution of EGFP, which probably obscured the distribution of TDP-43-EGFP fusion protein. How the EGFP was produced from the construct is not clear but by immunostaining using an anti-TDP-43 antibody, TDP-43 was predominantly nuclear in cells transfected with construct 1 (Fig. 3B), thus indicating that the TDP-43-EGFP fusion protein is predominantly distributed in nucleus, consistent with the literature [33], [34].

### TDP-43 fragments impair neurite growth in an aggregation-dependent or independent manner

**Figure 4:**
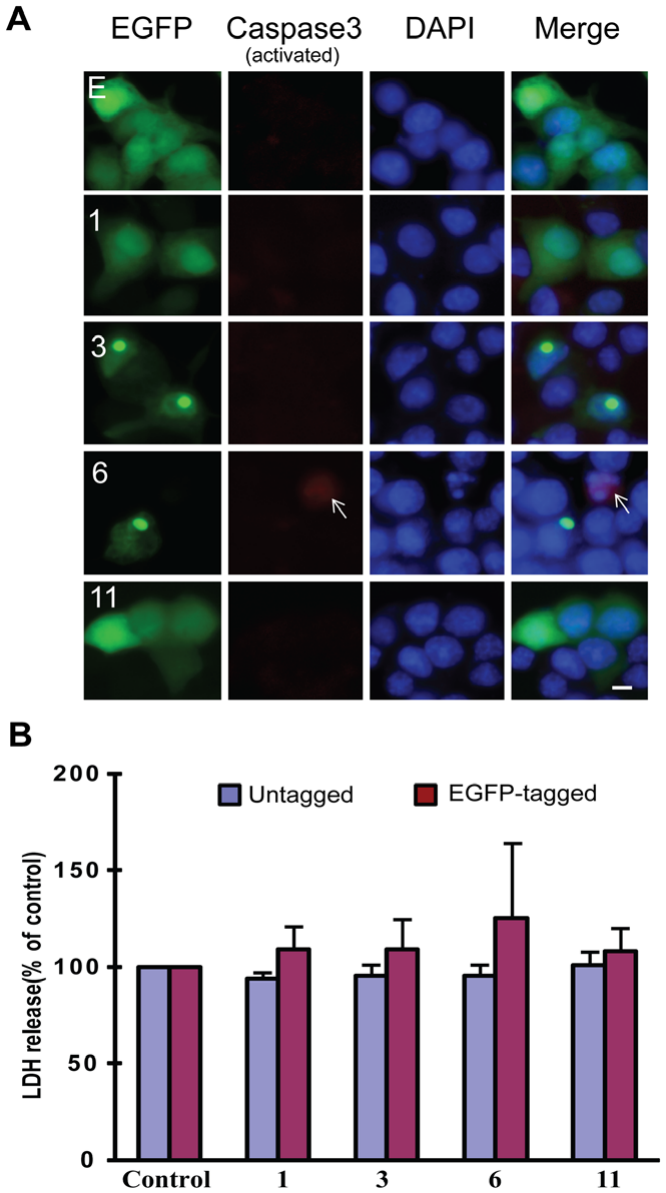
TDP-43 fragments did not increase cell death. (**A**) Caspase 3 activation was not associated with the expression of TDP-43 fragments. NSC-34 cells were transfected with the EGFP-tagged constructs (see Fig. 1) for 72 hrs. The cells were fixed and stained for activated caspase 3 (red). The numbers in each row indicate the constructs. DAPI stain marks the nucleus. The scale bar represents 10 µm. Notice that cells with caspase 3 activation could be observed (arrow) but they were not preferentially associated with the transfected cells. (**B**) LDH release was not significantly elevated in NSC-34 cells transfected with the TDP-43 fragments. Both untagged and EGFP-tagged TDP-43 constructs were transfected into NSC34 cells for 72 hrs. LDH activity was measured in the media and normalized to the activity in the carrier vector transfected control cells (set at 100%). The averages from three experiments were plotted. Error bars represent standard deviation. The numbers along the X-axis indicate the constructs.

To investigate the functional consequence of TDP-43 aggregation, we examined the cell death/viability. By several assays, including trypan blue staining, MTS assay, counting EGFP-fluorescent cells at 1, 2 and 3 days after transfection, TUNEL, activated caspase 3 staining and LDH release, we did not find a significant difference among the cells transfected with the constructs expressing EGFP and various TDP-43 fragments (Fig. 4 and data not shown), suggesting that the TDP-43 fragments, including those that form aggregates, do not significantly affect the viability of the cells in our culture.

**Figure 5:**
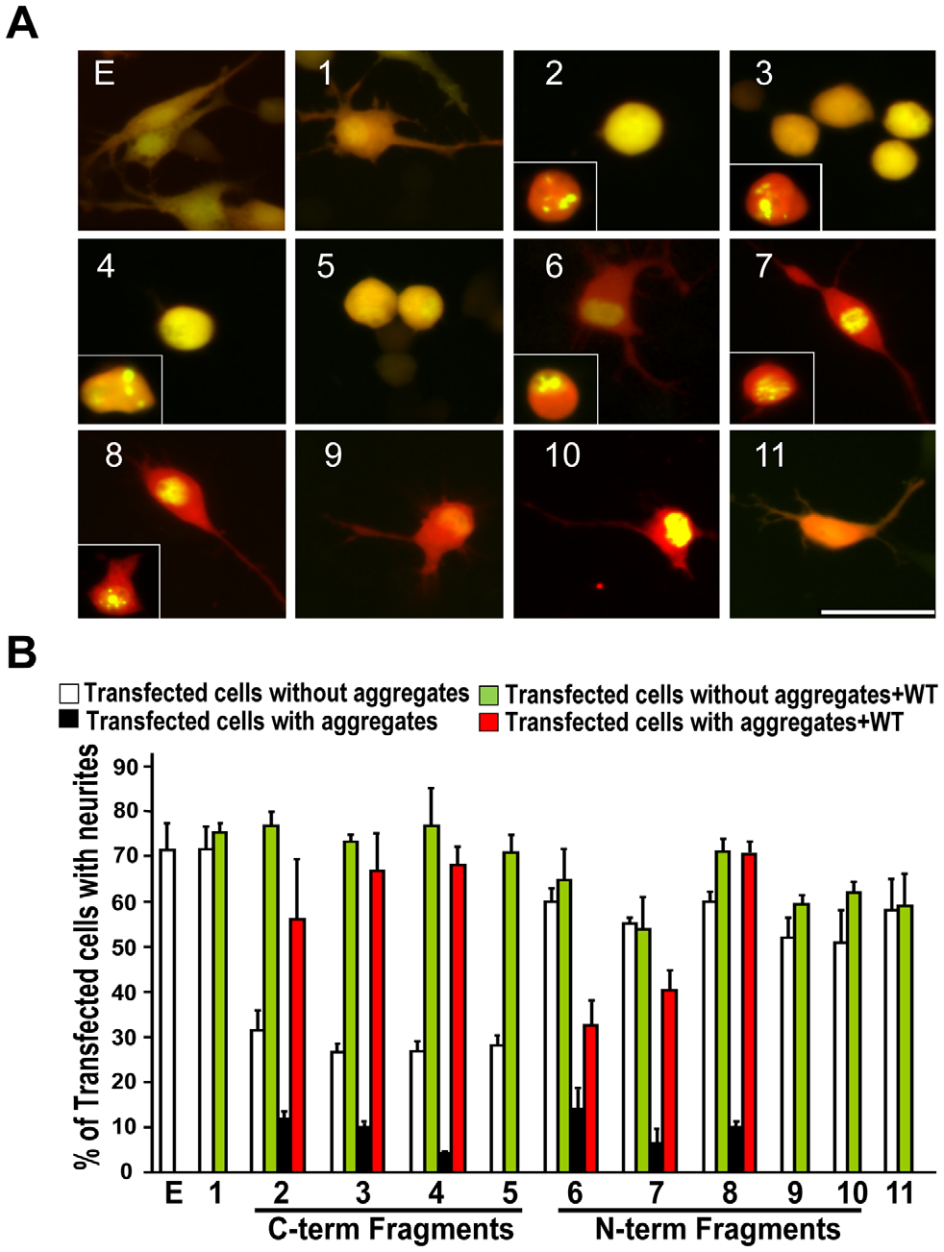
Expression of TDP-43 fragments inhibits neurite growth in motor neuron-like NSC-34 cells. (**A**) The cells were transfected with EGFP and each of the TDP-43 constructs as shown in Fig. 1. The cells were also transfected with tdTomato to help visualizing neurites. Twenty-four hours after the transfection, the cells were differentiated to neurons by serum withdraw and cultured for another 72 hours before being fixed and photographed. (**B**) TDP-43-EGFP fragments were transfected with either tdTomato (white and black bars) or with the full-length TDP-43 tagged with tdTomato (green and red bars; also see Fig. 9). Percent of cells bearing neurites were quantified and averaged from three experiments. Error bars represent standard error. Statistics using one-factor ANOVA showed significant difference among the cells transfected with different constructs (F = 24.6, p = 2×10^−27^). Tukey's Post Hoc Test showed significant differences comparing the EGFP-transfected cells (E) with the white bars of constructs 2, 3, 4 and 5 and the black bars of constructs 2, 3, 4, 6, 7 and 8 with (p<0.01).

**Figure 6:**
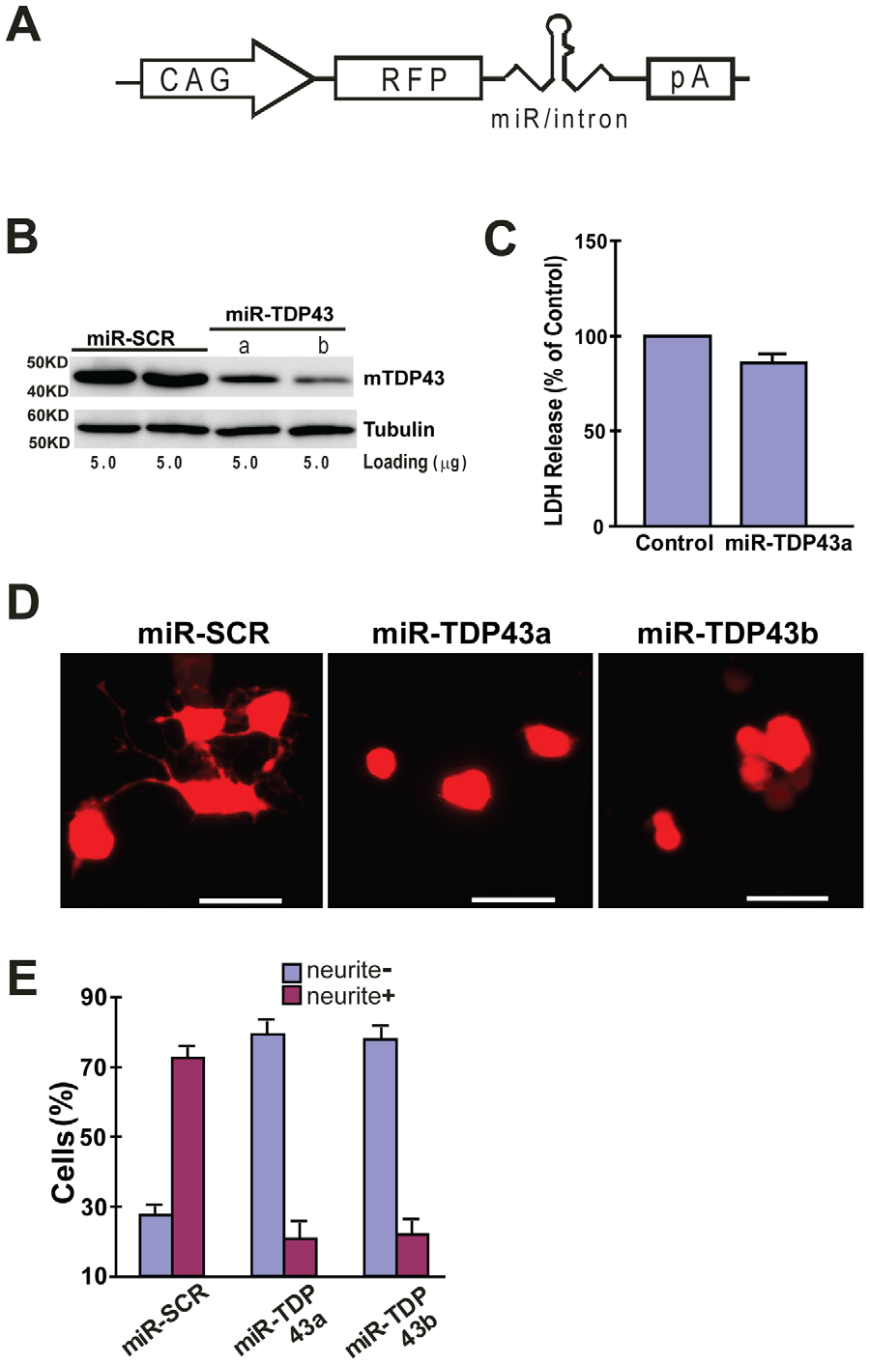
Knockdown of TDP43 inhibits neurite growth during neuronal differentiation of NSC-34 cells. (**A**) The miRNA expression construct, which uses the CAG promoter to drive expression of RFP (dsRed2) and a miRNA in an intron placed in the 3′-UTR of the RFP gene. The miRNA targets mouse TDP-43. (**B**) Western blot of proteins extracted from the transfected NSC-34 cells. TDP-43 was knocked down in cells transfected with two different miRNA constructs. Tubulin was detected as a loading control. miR-SCR is a scrambled miRNA that does not target any specific genes. (**C**) Knockdown TDP43 does not increase cell death. Scrambled or miR-TDP43a constructs were transfected into NSC34 cells. LDH activity was assayed in the media was 72 hrs after transfection. (**D**) Knockdown of TDP43 inhibits neurite growth in NSC34 cells. NSC34 cells were transfected with the miRNA constructs. Forty-eight hours later, neuronal differentiation was initiated. After another 72 hours, the cells were fixed and visualized by fluorescence microscopy. Scale bar represents 20 µm. (**E**) Quantification of cells with or without neurites. Results are shown as average percentage of cells from three independent experiments. Error bars are standard error. Statistics using one-factor ANOVA indicated significant difference among the cells transfected with different miRNA constructs (p = 0.0001). Tukey's Post Hoc Test showed significant differences comparing the miR-SRC-transfected cells with the cells transfected with either miR-TDP43s (p<0.01).

To determine whether the fragmented TDP-43 impairs neuronal characteristics, we transfected the constructs into NSC-34 cells and then induced neuronal differentiation. The cells transfected with full-length TDP-43-EGFP grew long neurites that were not different from the cells transfected with EGFP (Fig. 5, EGFP, construct 1). In contrast, cells transfected with the C-terminal TDP-43 fragments grew short or no neurites (Fig. 5, constructs 2–5), indicating that the C-terminal fragments impair neurite growth. Furthermore, this impairing activity was not dependent on TDP-43 aggregation. Both cells with aggregates (Fig. 5A, construct 2–4 inserts) or without (Fig. 6A, constructs 2–4) showed short or no neurites (Fig. 5B, white and black bars). Furthermore, construct 5, which had almost no aggregates (Table 1) and did not form any observable aggregates under the differentiation condition, strongly impaired neurite growth (Fig. 6). These observations indicate that aggregation is not a prerequisite for the C-terminal fragments to impair neurite growth.

In contrast to the C-terminal fragments, the majority of cells transfected with the N-terminal fragments or the middle fragment grew nearly normal neurites (Fig. 5A, constructs 6-11; Fig. 5B, white bars). However, a small number of cells that were transfected with constructs 6, 7 and 8 displayed aggregates (Fig 5A, constructs 6–8 inserts). In these cells, neurite growth was impaired (Fig. 5B, black bars), suggesting that for these N-terminal fragments the impairing activity for neurite growth depends on their aggregation.

### Overexpression of full-length TDP-43 rescues neurite growth

The TDP-43 fragments could impair neurite growth by a gain of toxicity that is independent of the normal TDP-43 function or by dominant negatively interfering with the normal TDP-43 function. If a gain of toxicity is true, overexpression of full length wild type TDP-43 should not affect this toxicity. On the other hand, if the dominant negative mechanism is true, overexpression of the wild type TDP-43 should compensate for the functional inhibition caused by the fragments and rescue neurite growth. To differentiate these two possibilities, we cotransfected the full-length TDP-43 with the TDP-43 fragments into the NSC-34 cells and quantified the number of transfected cells with neurites (Fig. 5B, green and red bars). The full-length TDP-43 was able to rescue the neurite growth from the inhibition caused by TDP-43 fragments. These results are consistent with the possibility that the TDP-43 fragments impair neurite growth by dominant negatively interfering with the normal function of TDP-43.

### TDP-43 function is required for neurite growth during neuronal differentiation

If interference of the normal function of TDP-43 caused impairment in neurite growth, then a loss of TDP-43 function is expected to have the same effect. To test this, we transfected NSC-34 cells with a microRNA (miRNA)-expression construct that silences TDP-43 expression. This construct employed the CAG promoter to drive expression of a primary miRNA, which encode a red fluorescent protein (dsRed2) and a pre-miRNA targeting mouse TDP-43 (Fig. 6A) [35]. Therefore, these constructs expressed both dsRed2 and a miRNA, which allowed us to track the transfected cells. To control for possible non-specific effects, such as stress associated with transfection, non-specific silencing or non-specific cellular responses to particular miRNA sequences, we used constructs that express two different miRNAs (miR-TDP-43a and miR-TDP-43b) targeting different regions in mouse TDP-43 mRNA. In addition, a control construct that expresses miRNA with scrambled sequence (miR-SCR) was used. We first tested whether the miRNA knocked down TDP-43 by transfecting these constructs into the NSC-34 cells. After 48 hours, we selected red-fluorescent cells using FACS and carried out Western blot on the protein extracted from the fluorescent cells. TDP-43 was knocked down in cells transfected with either TDP-43-specific miRNA compared with those cells transfected with the scrambled miRNA (Fig. 6B). Similar to the expression of TDP-43 fragments, TDP-43 knockdown did not have a significant effect on cell death/viability (Fig. 6C and data not shown) but significantly impaired the neurite growth (Fig. 6D, E).

### Confirmation of the observations in primary neurons

**Figure 7:**
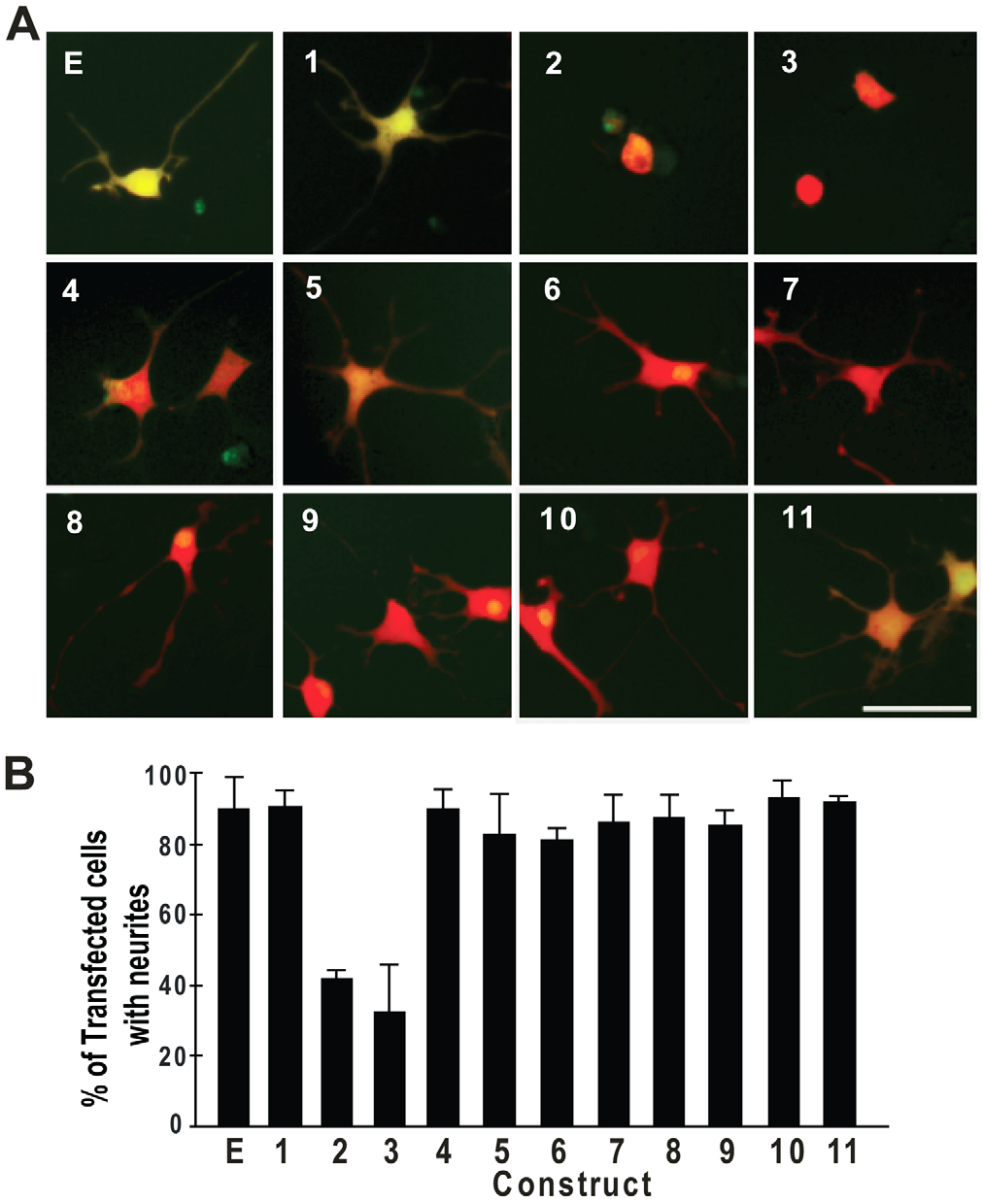
Expression of TDP-43 fragments inhibits neurite growth in primary rat forebrain neurons. (**A**) The rat forebrain neural precursors were transfected with EGFP and each of the TDP-43 constructs as shown in Fig. 1. The cells were also transfected with tdTomato for visualizing neurites. Twenty-four hours after the transfection, the cells were differentiated to neurons by withdrawing basic fibroblast growth factor 2 from the medium and cultured for another 72 hours before being fixed and photographed. (**B**) Quantification of neurite-bearing cells as shown in (A). Values are averages of four independent experiments. Error bars represent standard error.

To confirm the neurite phenotype observed in NSC-34 cells, we transfected the constructs into the rat forebrain neural precursors and then differentiated them into neurons. The neurons transfected with full-length TDP-43-EGFP, the N-terminal fragments (construct 6-10) and the RRM2 fragment (construct 11) grew normal neurites that were not different from the cells transfected with EGFP alone (Fig. 7). In contrast, cells transfected with two C-terminal TDP-43 fragments (construct 2 and 3) grew short or no neurites (Fig. 7). Differing from the NSC-34 cells, the C-terminal fragments 4 and 5 and the N-terminal fragments 6, 7 and 8 did not impair neurite growth in primary neurons (Fig. 7). This was most likely due to the relatively low transfection rate and the low expression levels achieved in the primary neurons, where no constructs formed aggregates. However, the two C-terminal fragments 2 and 3 impaired the neurite growth, suggesting that these two fragments have relatively potent toxicity.

**Figure 8:**
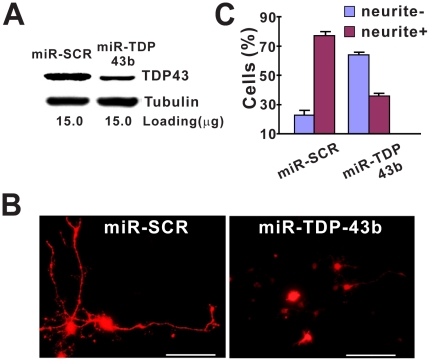
Knockdown of TDP43 inhibits neurite growth in differentiated primary rat fore brain neurons. (**A**) Western blot of proteins extracted from the transfected Rat-2 cells. TDP-43 was knocked down in cells transfected with miR-TDP43b. α-Tubulin was detected as a loading control. (**B**) Knockdown of TDP43 inhibits neurite growth in rat fore brain neurons. The cells were transfected with the miRNA construct for 48 hours and then differentiated. After another 72 hours, the cells were fixed and visualized by fluorescence microscopy. Scrambled miRNA (miR-SCR) was the control. Scale bar represents 100 µm. (**C**) Quantification of cells with or without neurites. Results are shown as average percentage of cells from three independent experiments. Error bars are standard error. Statistics using one-factor ANOVA indicated significant difference between the cells transfected with miR-SCR and those with miR-TDP43b (p = 0.00002).

To determine whether TDP-43 is required for neurite growth in primary neurons, we also conducted the knockdown experiments. Because the transfection efficiency was low, we first tested whether our two miRNAs (which were designed to silence mouse TDP-43) could knockdown rat TDP-43 by transfecting our constructs into rat-2 cells, which can be transfected with above 90% efficiency. Although both miR-TDP43a and miR-TDP43b matched rat TDP-43 mRNA sequence well, miR-TDP-43a did not knockdown rat TDP-43 (data not shown), probably due to the sequence differences in the regions flanking the miRNA target between the rat and mouse TDP-43 mRNA. miR-TDP43b knocked down rat TDP-43 efficiently (Fig. 8A). Therefore, we transfected miR-TDP-43b and miR-SCR into the rat primary neurons and found that neurite growth in cells transfected with miR-TDP-43b was also significantly impaired (Fig. 8B, C). These results confirm that TDP-43 also is required for neuronal differentiation in primary neurons.

### Fragmented TDP-43 co-aggregate with the wild type TDP-43 and reduce nuclear TDP-43

**Figure 9:**
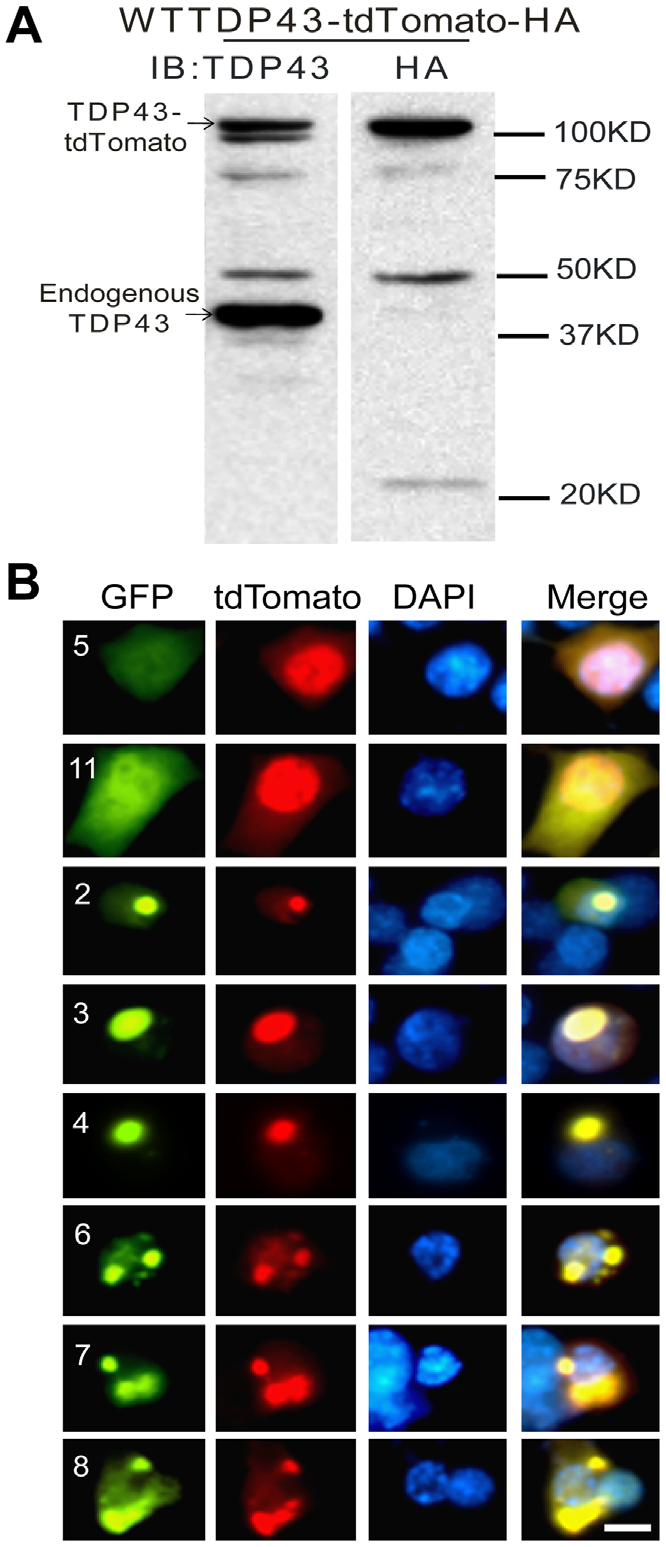
Coaggregation of wild type TDP-43 with its fragments. (**A**) The full-length TDP-43-tdTomato construct expressed the fusion protein. Proteins were extracted 48 hrs after the transfection of NSC-34 cells. Fifteen µg of the protein was resolved by SDS-PAGE and blotted. TDP-43-tdTomato was detected using a TDP43 antibody or an HA-tag antibody. (**B**) The EGFP-tagged, aggregation-prone TDP-43 fragments (**2**, **3**, **4**, **6**, **7** and **8**) were cotransfected with the full-length TDP-43-tdTomato construct. The full-length TDP-43 was colocalized with all of these mutants in aggregates. Two mutants (**5** and **11**) that do not form aggregates were also cotransfected with the full-length TDP-43. In these cells, the mutants (green) were diffusely distributed throughout the cells and the full-length wild type (red) was predominantly in the nucleus.

The above data are consistent with the hypothesis that TDP-43 fragments that contain RRM2 are prone to aggregation and impairing neurite growth by dominant-negatively interfering with the normal TDP-43 function. The functional impairment could be mediated through the interaction between the TDP-43 fragments and the full-length TDP-43. To test this possibility, we cotransfected the EGFP-tagged TDP-43 fragments with a full-length TDP-43 that was tagged with a red fluorescent protein tdTomato (RFP). Unlike the EGFP-tagged TDP-43, this construct expressed the full-length fusion protein and unfused tdTomato was detected (Fig. 9A). When cotransfected with the mutants that do not form aggregates (Fig. 9B, constructs 5 and 11), this wild type TDP-43 was predominantly in the nucleus with a diffused pattern. In contrast, when cotransfected with mutants that form aggregates, the wild type TDP-43 colocalized with the aggregates and the diffused TDP-43 signal in the nucleus was reduced (Fig. 9B, compare the red signal of constructs 2, 3, 4, 6, 7 and 8 with that of constructs 5 and 11). This suggests that the TDP-43 fragments can co-aggregate with the wild type TDP-43 and consequently deplete the TDP-43 in the nucleus.

**Figure 10 pone-0015878-g010:**
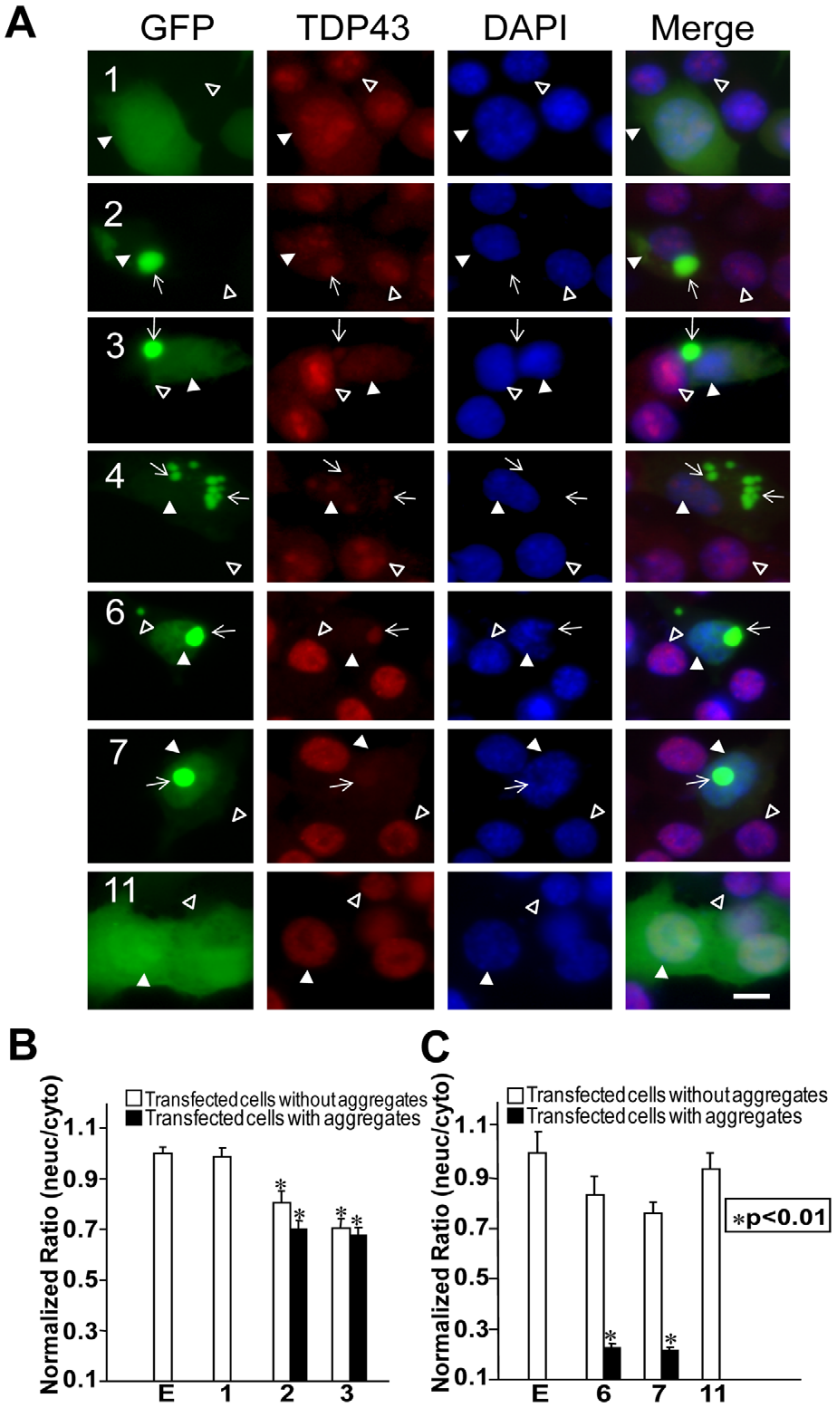
Expression of truncated TDP43 proteins reduces endogenous TDP-43 in the nucleus. NSC-34 cells were transfected with the constructs expressing the EGFP-tagged full length and truncated TDP-43 (see Fig. 1) as indicated by the numbers in each row of panels. Seventy two hours after the transfection, the cells were fixed and stained with antibodies against the N-terminal (for constructs 1, 2, 3, 4) or C-terminal peptides (for constructs 6, 7 and 11) of TDP-43 to visualize the endogenous TDP-43. The cells with aggregates (green, arrows) showed reduced staining nuclear TDP43 (red, filled arrowheads) compared with the untransfected cells (open arrowheads). Scale bar marks 10 µm. (B) Density ratio of nuclear to cytoplasmic TDP43 in cells stained by the antibody against the N-terminus of TDP-43. The construct 4 in A was not quantified because the number of transfected cells was too few to be reliable. (C) Density ratio of nuclear to cytoplasmic TDP43 in cells stained by the antibody against the C-terminus of TDP-43. The ratios were normalized against the average from the cells transfected by the EGFP construct. Each bar represents values averaged from between 18 to 28 cells. Error bars are standard error. The ratios from the cells transfected with the TDP-43 constructs were compared with the ratio from the cells transfected with the EGFP construct using student's t test with Bonferroni correction. The stars indicate p<0.01. Bars without stars indicate p>0.05.

To determine whether the aggregation reduces the endogenous nuclear TDP-43, we transfected the cells with the EGFP-tagged fragments. To avoid the interference from the transfected TDP-43 fragments, we observed the endogenous TDP-43 using antibodies against the N- or C-terminal peptide. In cells transfected with wild type TDP-43, there was an increase in the nuclear TDP-43 staining compared with the untransfected cells (Fig. 10A, #1, compare the nuclei pointed with filled arrowheads with those with the open arrowheads) or EGFP-transfected cells (not shown), but the overall ratio between the nuclear and cytoplasmic TDP-43 remained the same as the EGFP-transfected cells (Fig. 10B), indicating that both nuclear and cytoplasmic TDP-43 are increased to a similar extent. Similarly, in cells transfected with a fragment that does not form aggregates (construct 11), the TDP-43 staining intensity was the same as the untransfected cells (Fig. 10A, #11) or EGFP-transfected cells (Fig. 10C), indicating that this construct does not alter the nuclear and cytoplasmic distribution of endogenous TDP-43. In contrast, in cells transfected with the aggregation-prone fragments, the endogenous TDP-43 was detected in the aggregates (Fig. 10A, #2, 3, 4, 6 and 7, arrows) but was reduced in the nucleus (Fig. 10A, #2, 3, 4, 6 and 7, compare the nuclei pointed with filled arrowheads with those with open arrowheads). This observation was confirmed by quantification of the ratio between the nuclear and cytoplasmic staining density (Fig. 10B, C). Interestingly, in cells transfected with the C-terminal fragments, the ratio between the nuclear and the cytoplasmic TDP-43 was significantly reduced regardless whether the cells had aggregates or not (Fig. 10B). On the other hand, in cells transfected with the N-terminal fragments, this nuclear to cytoplasmic ratio was significantly reduced in cells with aggregates but remained unchanged in the cells without aggregates. Thus, the reduction of nuclear TDP-43 (Fig. 10) correlates with the inhibition of neurite growth (Fig. 5). These results suggest that these TDP-43 fragments can co-aggregate with the wild type TDP-43 and reduce the functional TDP-43.

### Untagged TDP-43 fragments behave similarly as the tagged fragments

**Figure 11:**
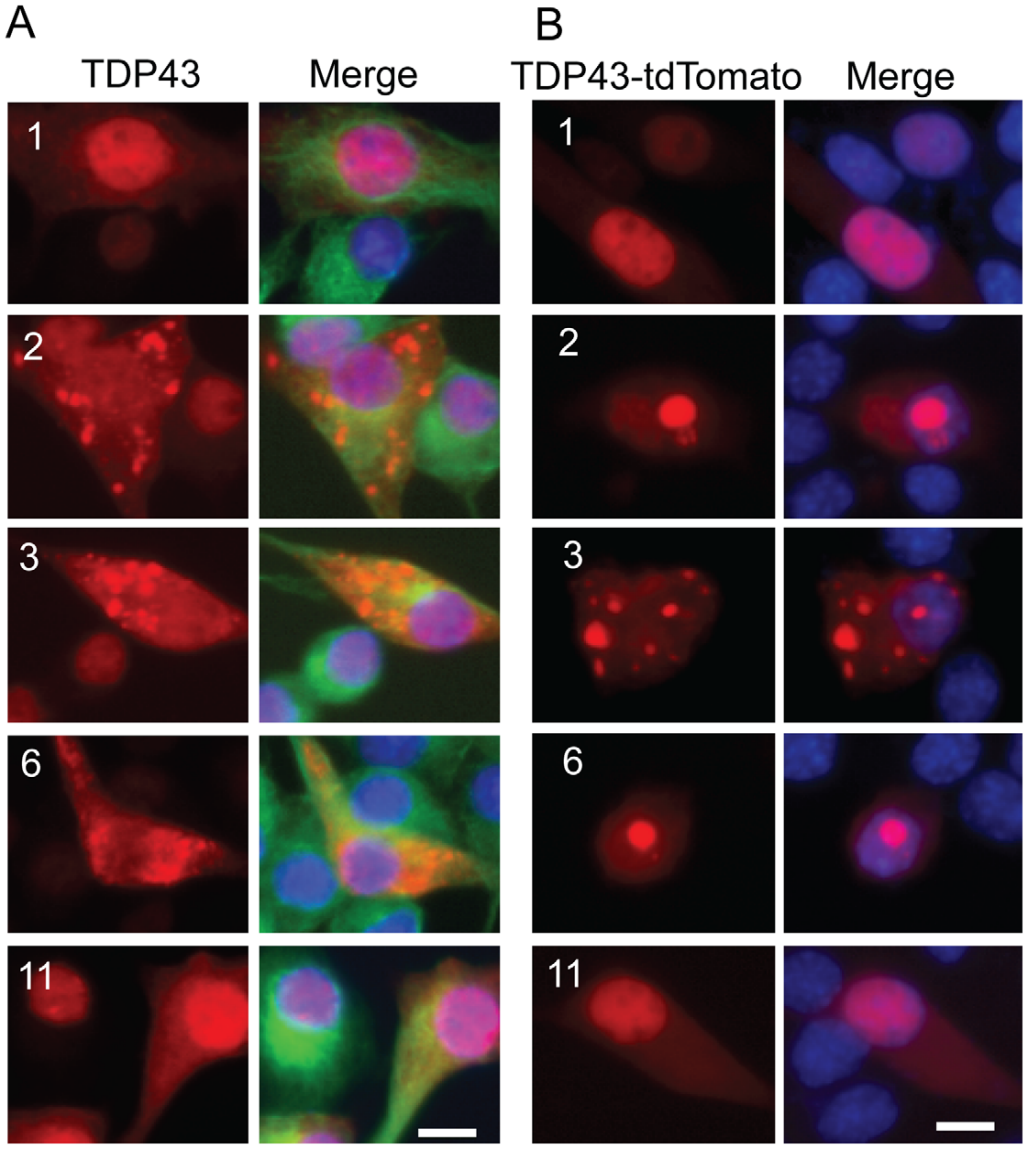
Untagged TDP-43 fragments form aggregates and coaggregate with the full-length TDP-43. (**A**) Untagged TDP-43 constructs were transfected into NSC-34 cells for 72 hrs. The cells were fixed and stained with TDP-43 (red) and tubulin (green) antibodies. The nucleus was revealed by DAPI staining. Notice that the construct 1 (full-length) and construct 11 did not form aggregates whereas constructs 2, 3 and 6 formed aggregates. (**B**) Untagged TDP-43 constructs were co-transfected with the tdTomato-tagged full-length TDP-43 for 72 hrs and then fixed. Notice that constructs 1 and 11 did not alter the distribution of the full-length TDP-43 whereas construct 2, 3, and 6 induced aggregation of the full-length TDP-43 and reduced the diffused staining of TDP-43 in the nucleus.

**Figure 12:**
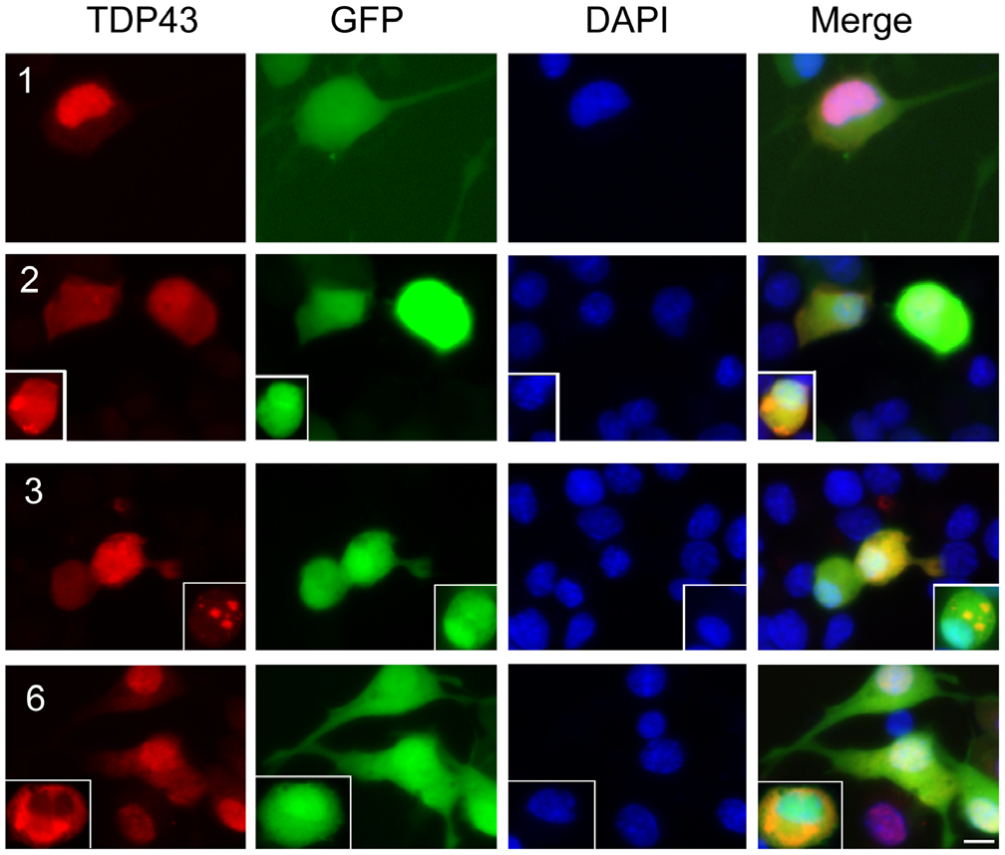
Untagged TDP-43 impaired neurite growth during neuronal differentiation. Untagged TDP-43 constructs were cotransfected with EGFP into NSC-34 cells for 24 hrs. The cells were differentiated for 72 hrs and fixed. Notice that cells transfected with the full-length TDP-43 grow long neurites whereas cells transfected with constructs 2 and 3 showed impaired neurite growth regardless whether the cell had aggregates (inserts) or not. Cells transfected with construct 6 showed impaired neurite growth only when aggregates were formed (insert).

To rule out the possibility that the above observations were caused by the tags, we constructed untagged constructs and transfected them into NSC-34 cells. Like the tagged constructs, constructs 1 (the full-length TDP-43) and 11 did not form aggregates whereas the aggregation-prone constructs (#2, 3 and 6) formed aggregates (Fig. 11A). Furthermore, when cotransfected with the full-length TDP-43, the aggregation-prone fragments induced aggregation of the full-length TDP-43 and reduced the diffused TDP-43 staining in the nucleus (Fig. 11B). Finally, expression of the untagged aggregation-prone constructs impaired neurite growth (Fig. 12). These results confirm the observations on the tagged TDP-43 fragments.

### Altering splicing is not a unifying property of the TDP-43 fragments that impaired neurite growth

**Figure 13:**
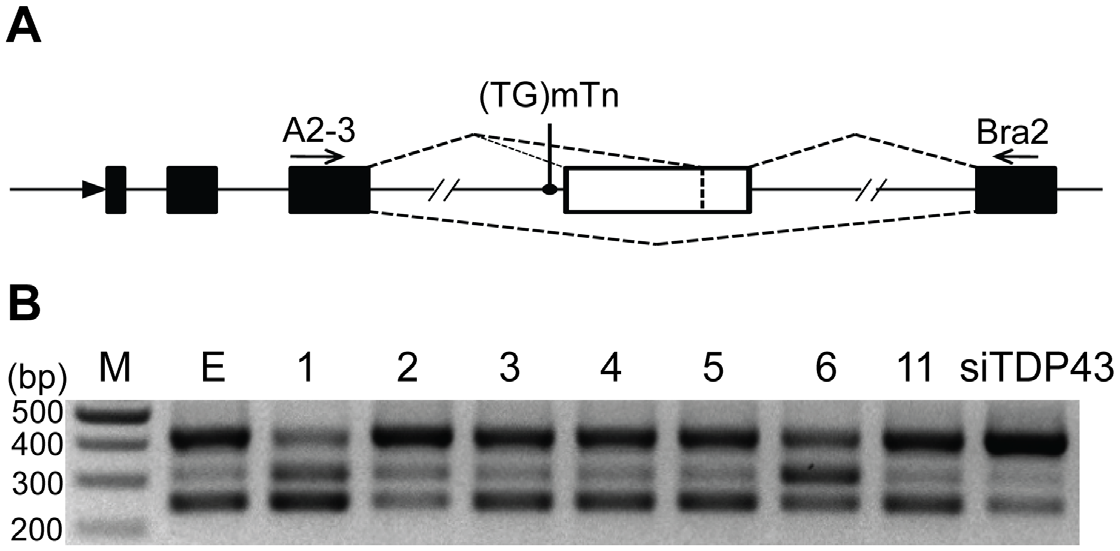
Effects of TDP43 fragments on CFTR splicing. (**A**) A schematic illustration of the CFTR reporter construct and its splicing products. The large arrow represents site of the promoter, black boxes represent non-CFTR exons, the white box represent CFTR exon, and small arrows depict primers used for amplification [53]. (**B**) HEK293 cells were transfected with full length or truncated TDP-43 constructs and (TG)13T3 CFTR reporter. The reporter transcript was detected by PCR using the primers indicated in (A). Top and lower bands correspond to the complete exon 9 (the white bar in A) inclusion and exclusion, respectively. The middle band corresponds to the splicing product from a cryptic 3′ splicing site (represented by the dashed line near the 3′ end of the white bar in A). As a control, TDP43 siRNA (siTDP43) was transfected. The numbers on the top of the lane indicate the constructs and E represents EGFP vector only.

A well-known function of TDP-43 is modulation of mRNA splicing. A best characterized example is that it promotes exon 9 skipping during the CFTR mRNA splicing [12]. To test whether the TDP-43 fragments alter the spicing-modulation activity, we assayed the CFTR splicing after transfecting various TDP-43 fragments into cultured cells. As expected, full length TDP-43 (construct 1) promoted exon 9 skipping while knockdown of TDP-43 by an siRNA inhibited this activity (Fig. 13). Construct 3, 4, 5 and 11 did not noticeably affect the CFTR splicing, whereas construct 2 inhibited exon 9-skipping activity (similar to TDP-43 knockdown by siTDP43) and construct 6 promoted an aberrant splicing (Fig. 13). It is notable that both constructs that interfered with the splicing contained RRM1, suggesting that RRM1 is required for the TDP-43 fragments to affect splicing. These results indicate that some TDP-43 fragments are capable of altering the CFTR splicing activity. However, this alteration is insufficient to account for the neurite growth-impairing activity of the TDP-43 fragments.

## Discussion

Our data show that both the C-terminal and the N-terminal fragments of TDP-43 can form aggregates in mammalian cells as long as they contain the C-terminal end of the RRM2 (Fig. 1, 2, Table 1). The C-terminal fragments appear to have a higher propensity for aggregation than the N-terminal fragments (Table 1). In addition, the aggregation-prone fragments can impair neurite growth during neuronal differentiation. However, the N-terminal fragments impair neurite growth in an aggregation-dependent whereas the C-terminal fragments impair neurite growth in an aggregation-independent manner (Fig. 5, 6). An overexpression of the full-length TDP-43 rescues whereas a knockdown of TDP-43 reproduces this impairment (Figs. 5, 7, 8). The TDP-43 fragments can co-aggregate with the full-length TDP-43, thereby reducing TDP-43 concentration in the nucleus (Figs. 9, 10). Although these results are initially obtained using EGFP-tagged TDP-43 fragments, the aggregation properties and the activity of impairing neurite growth can be replicated by expression of the untagged TDP-43 fragments (Fig. 11, 12). Taken together, these results suggest a model whereby the C-terminal fragments exert a dominant-negative toxicity on the neurons.

Consistent with this model, in FTD and ALS patients, the C-terminal fragments are generated [18], [36]. Cells in the central nervous system (CNS) with TDP-43-positive aggregates show a reduced nuclear TDP-43 [18]. TDP-43 function is essential for the survival of animals and for stem cells. Gene deletion in mice and Drosophila causes embryonic lethality [3], [4], [5], [37], [38]. Conditional gene deletion in cultured embryonic stem cells causes cell death [39]. Thus, a loss of functional TDP-43 to the co-aggregation with the C-terminal fragments could cause cell degeneration and its associated clinical symptoms.

Our result that the RRM2 is necessary for TDP-43 aggregation agrees with the recent literature, where all fragments capable of forming aggregates in cells contain the RRM2 region [21], [22], [30], [31]. The structural analysis of this region has revealed features consistent with a high aggregation propensity. RRM2 is a β-sheet-rich region and capable of self-association and forming extremely stable dimers [40], reminiscent of some of the structural features of another protein that is involved in ALS, Cu, Zn superoxide dismutase (SOD1) [41].

The C-terminal half of TDP-43 is important for pathogenesis. In addition to the presence of the C-terminal fragments in the CNS tissues from the patients, most ALS-associated mutations are located in this region [1]. It has been reported that the mutants, either the full-length or the C-terminal fragments, have a higher aggregation propensity than the wild type TDP-43 [29], [30], [42]. The C-terminal region is unstructured and involved in direct interactions with other proteins, some of which have been identified [6], [8], [43]. The N-terminal half of TDP-43 encompasses the nuclear localization signal (NLS) and RRM1 (Fig. 1). The latter is mainly responsible for binding RNA [44]. This N-terminal half cannot form aggregates unless the fragment is extended to include RRM2 (Fig. 2, Table 1). Once aggregation-enabled, however, the N-terminal fragments can impair the neurite growth in an aggregation-dependent manner. This suggests that the aggregation-enabled N-terminal fragments impair neurite growth primarily by coaggregation with the full-length TDP-43, thereby reducing the functional TDP-43. The C-terminal fragments, on the other hand, could impair neurite growth in at least two ways. Similar to the N-terminal fragments, it can coaggregate with the full-length TDP-43 and reduce the overall TDP-43 function (Figs. 9–11). In addition, it can interact with the other partner proteins to form non-functional complexes. Indeed, a multi-protein complex has been isolated from cultured cells that contained the C-terminal fragment [8]. Thus, it is conceivable that the C-terminal fragments can impair neurite growth in an aggregation-independent manner.

Our data indicate that the neurite growth can be an in vitro readout for the toxicity of TDP-43 fragments. Observations from other groups support the relevance and the reliability of this assay. In Drosophila, a loss of TDP-43 function leads to a decrease in dendritic branching during the development of larva nervous system [45]. Similarly, a loss of TDP-43 function in neuro2a cells and in zebra fish inhibited neurite growth [46], [47]. Due to the absence of the in vivo data, a role of impaired neurite growth in the pathogenesis of mammalian brain remains to be established. Nevertheless, the impairment of neurite growth can be used as an in vitro assay for further investigating the effect of a loss of TDP-43 function on neurons.

While the data from our experiments and the literature are consistent with a dominant-negative effect of the C-terminal fragments in neurodegeneration, this does not preclude a role from other properties of TDP-43 in the pathogenesis. For example, numerous studies have shown that the full-length TDP-43 can drive aggregation and toxicity when TDP-43 is overexpressed in vitro and in vivo [21], [26], [42], [48], [49]. This is not inconsistent with the dominant-negative mechanism. Recent data has demonstrated that the level of TDP-43 is tightly regulated to maintain a steady level. In heterozygote knockout mice, where only one of the two alleles of the TDP-43 gene actively expresses TDP-43, the mRNA and protein levels remain unchanged [3], [4], [5]. Conversely, overexpression of exogenous TDP-43 down-regulates the endogenous TDP-43 [49]. The reason for this tight regulation may be understood in the context that TDP-43 interacts with multiple protein partners to form multiprotein complexes [6], [8], [43]. Either a lack of TDP-43 or an over-abundance of TDP-43 could form dysfunctional complexes. The lack of TDP-43 is easily understood. The over-abundance of TDP-43 can form complexes composed of incomplete repertoire of the protein partners due to a limited supply of those partner protein, leading to dysfunctional complexes. For this reason, cells may have evolved mechanisms to ensure the proper levels of TDP-43. Therefore, overexpression of the wild type TDP-43 could cause dominant-negative diminution of TDP-43 function in several different ways, e.g. driving oligomerization and aggregation, elevating the production of the C-terminal fragments and increasing the formation of non-functional complexes.

In summary, our results suggest a model where the C-terminal fragments cause neuronal toxicity by a dominant-negative mechanism. This model does not preclude a role of toxicity from the other properties of TDP-43 protein and is consistent with the data in the current literature. Many details in this model remain hypothetical and will require data from experiments to confirm. Nevertheless, this model may be a useful guide for designing future experiments.

## Materials and Methods

### Vectors and cloning

To construct the full-length and truncated TDP-43 tagged with EGFP, the open reading frame (ORF) of human TDP-43 was amplified by PCR using the forward primers with XhoI restriction site at the 5′ end and the reverse primers with BamHI restriction site at the 5′ end. The PCR product was digested with XhoI and BamHI and inserted into the pEGFP-N1 Vector (BD Biosciences Clontech) that was digested using the same restriction enzymes. To construct the full-length TDP-43 tagged with tdTomato (kindly provided by Dr. Roger Tsien [50]), the TDP-43 ORF was amplified by PCR using the forward primer with HindIII restriction site at its 5′ end and the reverse primer with BamHI restriction site at the 5′ end. The PCR product was digested using HindIII and BamHI and inserted upstream of the tdTomato gene, which is in a pcDNA3.0 vector (Invitrogen). To construct the miRNAs for silencing TDP-43, the miRNAs were designed and generated base on the human miR-30a structure as previously described [51]. The target sequences are 5′-AAGGTGTTTGTTGGACGTTGT-3′ for miR-TDP43a and 5′-ATGCTGAACCTAAGCATAA-3′ for miR-TDP43b. The sense and antisense strands of DNA encoding the miRNAs were synthesized, annealed and digested with KpnI and XhoI. They were then cloned into an intron in the 3′ untranslated region of the dsRed2 gene [35], which is driven by the CAG promoter [52]. To construct untagged TDP43, the open reading frame (ORF) of human TDP-43 was amplified by PCR using a forward primer with EcoRV site at the 5′ end and a reverse primer with MluI site at the 5′ end. The PCR product was digested with EcoRV and MluI and inserted into the pCAG-RFP-miR [35], replacing the RFP. The pre-miRNA coding sequence was also removed from this plasmid. All constructs were sequenced for verification.

### Cell culture, transfection and neuronal differentiation

NSC-34 cells [32] were grown at 37°C in DMEM with 10% FBS. The cells were then transfected with constructs using Lipofectamine 2000 according to manufacturer's recommendations (Invitrogen). For neuronal differentiation, twenty four (for TDP43 and its fragments) or 72 hours (for miRNA constructs) after transfection, the cells were transferred to wells coated with poly-L-lysine and cultured at a density of 2×10^3^ cells/cm^2^ in the medium consisting of 1∶1 DMEM and Ham's F12 (Gibco BRL) supplemented with 1% FBS, 100 U/mL penicillin, 100 µg/mL streptomycin, and 2 mM glutamine. After 72 hrs, the cells were fixed using 4% paraformaldehyde and 0.1% glutaraldehyde in PBS.

For primary neuronal cultures, neural precursors were prepared from forebrain of embryonic day 14.5 (E14.5) SASCO SD rat embryos (Charles River, Wilmington, MA). Cells were plated out at 1.2–1.4×10^6^ cells per 100-mm dishes precoated with 15 µg/mL poly-L-ornithine (Sigma) and 1 µg/mL fibronectin (R&D Systems, Minneapolis, MN), and treated daily with 10 ng/mL of basic fibroblast growth factor 2 (FGF2) (R&D Systems). At 5 d *in vitro*, cells were passaged and plated out at 0.8–1.0×10^6^ cells per precoated plate and expanded as above for an additional 3–4 d (expansion, ∼300%). Cells were passaged again and plated out at 1.2–1.4×10^6^ per precoated plate. The cells were then transfected with constructs using Lipofectamine 2000 (Invitrogen). Two days after transfection, FGF2 was removed to induce neuronal differentiation. After another 2 days, cells were washed once with PBS and fixed.

HeLa cells (ATCC) were grown at 37°C in DMEM/10% FBS. The cells were then transfected with constructs using Lipofectamine 2000 (Invitrogen), cultured for 48 hours and then fixed.

### Cell viability/death assays

NSC34 cells were transfected with different constructs and 72 hours after transfection, cell viability/death assays were performed. Terminal deoxynucleotidyl transferase dUTP nick end labeling (TUNEL) assays were performed using the In Situ Cell Death Detection kit, TMR red (Roche, Indianapolis) according to the manufacturer's instructions. The 3-(4,5-dimethylthiazol-2-yl)-5-(3-caboxymethoxyphenyl)-2-(4-sulfophenyl)-2H-tetrazolium (MTS)-based cell proliferation assay (MTS assay) was carried out using the CellTiter 96 Aqueous One Solution Cell Proliferation Assay (Promega, Madison, WI), according to the manufacturer's instructions. Absorbance at 490 nm was measured in a microplate reader (Benchmark, Bio-Rad Laboratories, inc). After 72 hours transfection, the medium was collected for NSC34 cells and a LDH assay (Promega) was used to measure LDH levels as an indicator of toxicity. Cells were then fixed and immunofluorescent staining was done using an antibody against activated caspase-3 (1∶200; Cell Signaling Technology) and an Alexa Fluor®568-conjugated goat anti-rabbit IgG (1∶400; Invitrogen).

### Immunofluorescence microscopy

After fixation for 15 min at room temperature, cells were permeabilized by using 0.5% Triton in PBS for 5 min. Nuclei were stained with 4-, 6-diamidino-2-phenylindole (DAPI). To stain for TDP43, the fixed cells were incubated in the blocking buffer (5% Heat-inactivated goat serum, 0.3% Triton-X-100, 5% fat-free dry milk in PBS, pH 7.4) for 15 min at room temperature and then incubated with a rabbit polyclonal antibody (ProteinTech Group, Inc, Chicago, IL) at 1∶100 dilution or N- or C-terminal rabbit polyclonal antibody (Kind gifts from Dr. Virginia Lee) at 1∶1000 dilution in the blocking buffer at 4°C. After overnight incubation, cells were washed and incubated with Alexa Fluor®568-conjugated goat anti-rabbit IgG (invitrogen) at room temperature for 1 hour. To stain Tubulin, a mouse monoclonal anti-α-tubulin (1∶100) and Alexa Fluor®488-conjugated goat anti-mouse IgG (invitrogen) were used.

The cells were visualized and photographed using a Nikon Eclipse Ti fluorescence microscope. For observation of nuclear aggregates, a series of optical sections were taken along the z-axis and processed by deconvolution (AutoQuant X, Media Cyberbetics) to ensure that the aggregates are within the bounds of the nucleus. For quantification of neurites, the cells with at least one neurite longer than 10 µm in NSC-34 cells or longer than 30 µm in primary neurons were counted as having neurites. Otherwise, the cells were counted as having no neurites. For illustration, some panels were adjusted for brightness and contrast as a whole.

The endogenous nuclear and cytoplasmic TDP-43 was estimated by fluorescence density measurement. When the cells were transfected with the full-length or the C-terminal fragments, the antibody against the N-terminus of TDP-43 was used for staining. When the cells were transfected with the N-terminal fragments, the antibody against the C-terminus of TDP-43 was used. After staining the cells were visualized and photographed. For each cell, average density of TDP43 staining was measured in the nucleus and the cytoplasm by Nikon NIS-Elements software. The density ratio of the nucleus to the cytoplasm was calculated and then normalized to the average ratio of the EGFP control.

### Immunoblotting

Cells were collected by a hard plastic cell scraper from the plates and centrifugation at 400 g. The pellets were resuspended in RIPA buffer (0.1% SDS, 1% Nonidet P-40, 0.5% sodium dexoycholate, 5 mM EDTA, 150 mM NaCl, 50 mM Tris-HCl, pH 8.0) plus protease inhibitors (Pierce). After clearing the lysate by centrifugation, protein concentration of cell lysates or tissue homogenates was measured using BCA assay (Pierce, Rockford, IL). The samples were heated in Laemmli's buffer, and equal amounts of protein were loaded and resolved by SDS-PAGE. After transfer to nitrocellulose membranes, blots were blocked with 5% nonfat dry milk in PBST (0.25% Triton X-100 in PBS, pH 7.4) for 1 h, and then incubated with primary antibodies overnight. After washing 3 times in PBST, the membrane was incubated with anti-rabbit IgG (1∶5000; Amersham Biosciences) or mouse IgG (1∶5000; invitrogen) conjugated to horseradish peroxidase for 1 hour. Membranes were washed three times and proteins were visualized after ECL (Pierce) treatment and detected using the LAS-3000 imaging system. The primary antibodies used were rabbit polyclonal anti-EGFP (1∶1000, Clontech) and anti-TDP-43 (1∶1000; ProteinTech Group, Inc, Chicago, IL); and mouse monoclonal anti-α-tubulin (1∶1000; Sigma) and anti-HA (1∶1000, Roche).

### Analysis of CFTR mRNA splicing

The experiment was conducted based on a previously published method [53]. Briefly, HEK293 cells were first transfected with full length or truncated TDP-43 constructs. After 48 hrs, they were transfected with (TG)13T3 CFTR reporter plasmid. After another 24 hrs, cells were harvested and total RNA was isolated. RT-PCR was carried out using 5 µg of total RNA with forward A2-3 (5′-CAACTTCAAGCTCCTAAGCCACTGC-3′) and reverse Bra2 (5′-TAGGATCCGGTCACCAGGAAGTTGGTTAAATCA-3′) primers. PCR was performed at 95°C for 5 min, followed by 30 cycles of denaturing at 95°C for 30 s, annealing at 57°C for 30 s, extension at 72°C for 60 s. PCR products were visualized on agarose gels.
